# Severity and Determinants of Anemia Among Adolescent Girls in Africa: A Multicountry and Multilevel Analysis of 25 Countries

**DOI:** 10.1155/anem/1066665

**Published:** 2026-10-18

**Authors:** Bedilu Alamirie Ejigu, Getnet Bogale Begashaw, Fentanesh Nibret Tiruneh

**Affiliations:** ^1^ Department of Statistics Addis Ababa University Addis Ababa Ethiopia aau.edu.et; ^2^ Department of Data Science Debre Berhan University Debre Berhan Ethiopia dbu.edu.et; ^3^ Department of Human Nutrition Bahir Dar University P.O. Box 79 Bahir Dar Ethiopia bdu.edu.et; ^4^ Bahir Dar Food and Nutrition Research Center Bahir Dar University P.O. Box 79 Bahir Dar Ethiopia bdu.edu.et

**Keywords:** adolescent girls, Africa, anemia, health and nutrition

## Abstract

**Background:**

Anemia remains a major public health concern in Africa, with substantial health and developmental consequences. Adolescents, particularly girls, are at increased risk due to rapid growth, menstruation, and inadequate dietary intake. Identifying the severity and determinants of anemia among adolescent girls in Africa is crucial for developing appropriate interventions, yet evidence remains scarce.

**Objective:**

To assess the severity and determinants of anemia among adolescent girls in Africa using nationally representative data.

**Methods:**

Data were obtained from the most recent Demographic and Health Surveys (DHS) of 25 African countries, including 41,662 adolescent girls aged 15–19 years. The severity of anemia was determined using descriptive statistics based on proportions. Anemia severity was modeled as a four‐level ordinal outcome (none, mild, moderate, and severe) using survey‐weighted multilevel ordinal (proportional‐odds) logistic regression with a random intercept at the cluster level. Chi‐square tests assessed initial associations, and adjusted odds ratios (AOR) with 95% confidence intervals (CI) were reported from the final model.

**Results:**

The overall magnitude of anemia among adolescent girls was 42.3% (95% CI: 41.96–44.20). In the multivariable analysis, factors significantly associated with lower odds of anemia included primary education (AOR = 0.84; 95% CI: 0.75–0.94), secondary and above education (AOR = 0.86; 95% CI: 0.75–0.98), rich and richer wealth status (AOR = 0.77; 95% CI: 0.65–0.91), employment (0.86; 95% CI: 0.78–0.94), hormonal contraceptive use (AOR = 0.44; 95% CI: 0.38–0.51), parity (0.85; 95% CI: 0.76–0.95), and normal and overweight/obese nutritional status (AOR = 0.84; 95% CI:0.73–0.97 and AOR = 0.75; 95% CI: 0.62–0.91), respectively.

**Conclusion:**

Anemia affects a substantial proportion of adolescent girls in Africa and is influenced by educational, economic, reproductive, and nutritional factors. Interventions should focus on keeping girls in school, encouraging employment opportunities, and maintaining healthy bodyweight. Addressing these determinants is vital for improving adolescent girls’ health and achieving sustainable development goals.

## 1. Introduction

Anemia is a condition in which the number of red blood cells or hemoglobin is lower than normal, reducing the blood’s capacity to carry oxygen. It can result from several factors: nutrient deficiencies or poor nutrient absorption, infections (e.g., malaria, parasitic infections, tuberculosis, and HIV), inflammation, chronic diseases, gynecological, and obstetric conditions, and inherited red blood cell disorders. The most common cause of anemia is iron deficiency, which can occur due to inadequate dietary intake, poor iron absorption, or increased needs during growth spurts and menstruation [1].

Approximately one‐quarter of the global population is estimated to be anemic, with the prevalence notably higher among women, expectant mothers, adolescent girls, and children under the age of five. Anemia is the third‐leading cause of years lived with disability (YLDs) globally [2]. Between 1990 and 2021, the evidence indicates a global trend towards less severe anemia. The most significant reductions were observed in adult males; however, progress has been slower among women of reproductive age including adolescent girls aged 15–19 and children under the age of five. In 2021, sub‐Saharan Africa and South Asia are facing the greatest burden [3].

Adolescence is a period of transition from childhood to adulthood, characterized by rapid physical growth and increased nutritional needs. Among adolescents, especially in low‐ and middle‐income countries, anemia is a significant issue. During this stage, the body undergoes rapid growth and development, increasing nutritional demands, including for iron. Iron deficiency anemia adversely affects the educational and economic well‐being of adolescents. It has been linked to stunting, wasting, being underweight, poor cognitive function, reduced physical activity, and attention deficit hyperactivity disorder in adolescents [4, 5].

The onset of menstruation further raises the risk of anemia due to additional iron loss among adolescent girls. Adolescent girls are vulnerable, especially to iron deficiency due to the accelerated increase in requirements for iron, poor dietary intake of iron, menstrual losses, infection, the norm of early marriage, and adolescent pregnancy. Adolescents with anemia are more likely to experience anemia during pregnancy, which can lead to various adverse effects on both pregnancy and childbirth [6, 7].

Adolescent girls in Africa are particularly vulnerable to anemia due to a combination of factors. Inadequate diets and poor absorption of iron, compounded by high rates of infectious diseases like malaria and parasitic infections, and poor hygiene management, contribute significantly. Limited access to healthcare and education, along with poverty and gender inequality, exacerbate the issue. Rapid growth during adolescence increases nutritional needs, which are difficult to meet in regions with limited resources [8–12].

Studies have identified factors significantly associated with anemia among adolescent girls including the respondent’s age, marital status, educational level, nutritional status, and wealth status [7, 13, 14]. Additionally, early marriage and pregnancy, which are prevalent in many African countries, are linked to a range of social, physical, and health problems, including anemia among adolescents. Early marriage often leads to early pregnancy, which increases the risk of anemia due to higher iron requirements and inadequate nutritional intake. Moreover, adolescents who marry early may face limited access to healthcare, education, and economic opportunities, further exacerbating their vulnerability to anemia. These factors contribute to a cycle of poor health and socioeconomic disadvantage that can have long‐lasting effects on their overall well‐being and future prospects [15, 16].

Despite global efforts to mitigate anemia, progress has been slower among adolescent girls, especially in low‐ and middle‐income countries. The current trends in anemia make it challenging to achieve the Sustainable Development Goal (SDG) of reducing anemia among women of reproductive age by 50% by 2030 [17, 18]. Addressing all forms of malnutrition is a key target of the SDGs. By effectively reducing malnutrition, the prevalence of anemia can also be lowered, thereby contributing to the successful achievement of the SDGs by 2030. To meet this ambitious goal, comprehensive strategies must be implemented including assessment the severity and determinants of anemia among adolescent girls [19].

While studies have estimated the magnitude and determinants of anemia among women of reproductive age, less attention has been given to adolescent girls, and recent multicountry analyses are rare.

Our previous preprint study, conducted across 24 African countries and published on Research Square, reported an overall anemia prevalence of 43.6% among adolescent girls [20], using a dichotomous classification (anemic vs. nonanemic), which limited the ability to capture the full burden and clinical significance of the condition. The current revised study addresses this gap by classifying anemia into four categories: severe, moderate, mild, and nonanemic—thereby providing a more nuanced understanding of its distribution and public health implications. In addition, the study analyzed nationally representative data from 41,662 adolescent girls across 25 African countries, covering the most recent ten‐year period. The current study expands both the temporal and geographic scope, enabling identification of countries most affected by varying levels of anemia severity. This comprehensive multicountry analysis will generate critical evidence on the determinants and country variation of anemia severity, helping to inform targeted, context‐specific interventions, prioritize high‐burden settings, and recommend more efficient allocation of resources to improve adolescent girls’ health outcomes across the continent.

## 2. Methods

### 2.1. Data Source and Study Participants

Data from the most recent Demographic and Health Surveys (DHSs) from 25 African countries between 2016 and 2025 were used for this study. The countries were selected based on two criteria: (1) the availability of recent DHS datasets within the past ten years and (2) the availability of hemoglobin measurement data used to assess anemia status. Of the countries initially pooled from the DHS program, two were excluded because valid hemoglobin measurements were not available for adolescent girls (no or invalid anemia measurement), yielding a final set of 25 countries. The 25 included countries and their survey years are listed in Table 1.

**TABLE 1:** Severity of anemia among adolescent girls across African countries.

Country name	Survey year	Anemia status									
		Severe		Moderate		Mild		Not anemic		Total anemia	
		*n* (%)	95% CI	*n* (%)	95% CI	*n* (%)	95% CI	*n* (%)	95% CI	*n* (%)	95% CI
Benin	2017–18	39 (2.3)	1.6–3.2	487 (29.5)	27.0–32.2	426 (25.6)	23.4–27.9	693 (42.6)	39.6–45.6	952 (57.4)	54.4–60.4
Burkina Faso	2021	19 (1.2)	0.7–1.9	548 (29.8)	26.9–32.9	484 (26.7)	24.3–29.1	817 (42.4)	39.2–45.6	1051 (57.6)	54.4–60.8
Burundi	2016–17	15 (0.9)	0.5–1.6	293 (16.0)	14.0–18.3	358 (19.0)	17.0–21.1	1288 (64.1)	61.2–66.9	666 (35.9)	33.1–38.8
Cameroon	2018–19	21 (0.9)	0.5–1.4	249 (16.8)	14.5–19.5	414 (24.0)	21.6–26.6	972 (58.3)	55.0–61.5	684 (41.7)	38.5–45.0
Côte d’Ivoire	2021	20 (1.0)	0.6–1.6	425 (31.3)	27.7–35.2	429 (30.0)	26.8–33.3	564 (37.8)	34.6–41.0	874 (62.2)	59.0–65.4
DR Congo	2023–24	25 (1.3)	0.7–2.2	619 (16.9)	14.9–19.2	770 (22.3)	20.1–24.7	1980 (59.5)	56.4–62.6	1414 (40.5)	37.4–43.6
Ethiopia	2016	44 (0.9)	0.5–1.5	315 (7.5)	6.2–9.1	396 (11.5)	10.0–13.2	2417 (80.1)	77.8–82.2	755 (19.9)	17.8–22.2
Gabon	2019–21	38 (3.5)	2.1–5.7	432 (36.2)	32.0–40.7	368 (32.3)	28.5–36.3	369 (28.0)	24.1–32.3	838 (72.0)	67.7–75.9
Gambia	2019–20	20 (1.0)	0.6–1.7	259 (16.1)	13.2–19.4	351 (26.4)	23.4–29.6	701 (56.5)	52.3–60.7	630 (43.5)	39.3–47.7
Ghana	2022–23	13 (1.6)	0.8–2.9	293 (19.7)	17.2–22.4	345 (22.6)	19.9–25.6	773 (56.2)	52.8–59.5	651 (43.8)	40.5–47.2
Guinea	2018	18 (1.3)	0.8–2.2	276 (21.8)	19.3–24.5	288 (24.0)	21.7–26.5	666 (52.9)	49.8–56.0	582 (47.1)	44.0–50.2
Lesotho	2023–24	7 (1.1)	0.4–2.9	122 (17.2)	14.0–20.9	154 (24.8)	20.6–29.4	397 (56.9)	52.3–61.4	283 (43.1)	38.6–47.7
Liberia	2019–20	5 (0.7)	0.2–2.2	243 (29.6)	25.0–34.6	245 (24.8)	21.5–28.6	414 (44.9)	40.2–49.7	493 (55.1)	50.3–59.8
Madagascar	2021	15 (0.7)	0.3–1.3	277 (12.4)	10.8–14.2	330 (14.7)	12.9–16.6	1456 (72.2)	69.6–74.6	622 (27.8)	25.4–30.4
Malawi	2024	20 (1.1)	0.6–2.0	259 (12.8)	11.0–14.7	349 (17.8)	15.6–20.1	1408 (68.3)	65.6–70.9	628 (31.7)	29.1–34.4
Mali	2023–24	37 (1.5)	1.0–2.2	504 (24.4)	22.2–26.8	431 (23.5)	21.3–25.9	987 (50.6)	47.7–53.5	972 (49.4)	46.5–52.3
Mauritania	2019–21	115 (7.3)	5.7–9.2	567 (31.9)	28.9–35.1	381 (24.1)	21.3–27.2	653 (36.7)	33.1–40.5	1063 (63.3)	59.5–66.9
Mozambique	2022–23	21 (2.2)	1.3–3.8	318 (27.3)	24.1–30.8	344 (24.8)	21.8–28.2	707 (45.6)	42.1–49.2	683 (54.4)	50.8–57.9
Nigeria	2023–24	19 (0.8)	0.4–1.3	537 (17.9)	16.1–19.8	685 (23.5)	21.7–25.4	1601 (57.8)	55.2–60.4	1241 (42.2)	39.6–44.8
Rwanda	2019–20	1 (0.1)	0.0–0.5	65 (4.0)	3.1–5.1	170 (10.7)	9.2–12.3	1434 (85.3)	83.2–87.1	236 (14.7)	12.9–16.8
Sierra Leone	2019	27 (2.1)	1.4–3.2	402 (25.4)	23.0–28.0	355 (21.9)	19.8–24.1	767 (50.6)	47.6–53.6	784 (49.4)	46.4–52.4
South Africa	2016	7 (0.9)	0.4–2.0	90 (19.4)	15.0–24.6	75 (13.6)	10.6–17.4	343 (66.1)	61.1–70.8	172 (33.9)	29.2–38.9
Tanzania	2022	38 (1.8)	1.3–2.7	334 (21.1)	18.3–24.2	373 (22.0)	19.6–24.6	849 (55.0)	51.5–58.5	745 (45.0)	41.5–48.5
Uganda	2016	21 (1.6)	1.0–2.5	176 (12.9)	10.9–15.1	262 (18.4)	16.1–21.0	918 (67.1)	64.1–70.0	459 (32.9)	30.0–35.9
Zambia	2024	31 (1.7)	1.2–2.5	261 (15.7)	13.9–17.7	320 (19.1)	17.0–21.4	1027 (63.5)	60.7–66.1	612 (36.5)	33.9–39.3

*Note:* Values are weighted *n* (%) with 95% confidence intervals (CI). The total analytic sample was *N* = 41,662 across 25 countries; total anemia includes mild, moderate, and severe anemia. *n* = weighted frequency; *N* = total sample size.

Abbreviation: CI, confidence interval.

The DHS implements a stratified two‐stage cluster sampling design which gives nationally representative samples. The sampling design involved randomly selected communities (clusters) at the first stage and households at the second stage. Further details on the implementation of the DHS survey were available in other sources [21]. Anemia testing was performed after getting consent from the participants. Among reproductive‐age women, about 41,662 were adolescents (15–19 years). Consequently, the final analytical sample for this study consisted of 41,662 female adolescents.

### 2.2. Measurements

#### 2.2.1. Outcome Variable

Unlike our previous preprint work [20] which treats the outcome variable as binary (anemic vs. nonanemic), the outcome variable in the current study was anemia severity among adolescent girls, categorized into four levels: nonanemic (coded as “0”), mild anemia (coded as “1”), moderate anemia (coded as “2”), and severe anemia (coded as “3”). Anemia level was determined based on the women’s hemoglobin concentration, measured in grams per deciliter (g/dL), according to the cut‐off points defined by the World Health Organization (WHO). For pregnant women, anemia was defined as hemoglobin levels < 11 g/dL and further classified as mild anemia (10.0–10.9 g/dL), moderate anemia (7.0–9.9 g/dL), and severe anemia (< 7.0 g/dL). For nonpregnant women, anemia was defined as hemoglobin levels < 12 g/dL and categorized as mild anemia (11.0–11.9 g/dL), moderate anemia (8.0–10.9 g/dL), and severe anemia (< 8.0 g/dL) [22].

#### 2.2.2. Predictor Variables

The predictor variables included in this analysis were marital status (ever married and never married/single), age at first marriage (> 15 years old and 15–19 years old), educational level (no education, primary and secondary, or above), employment status (not employed and employed), place of residence (urban and rural), nutritional status (underweight, normal weight, and overweight/obese), pregnancy status (not pregnant and pregnant), history of childbearing/parity (no birth history and one or more birth history), contraceptive use (none user, hormonal user, and none hormonal user), use of mosquito nets (no/yes), and health insurance coverage (no/yes). The wealth index was constructed based on household ownership of selected assets, such as televisions and materials used for constructing the house, source of drinking water, type of toilet facility, etc. Household asset factor scores were generated using principal component analysis, then standardized and categorized into quintiles (poorer, poor, middle, rich, and richer).

### 2.3. Statistical Analysis

The complexity of analyzing DHS survey datasets can be attributed to two main factors: (i) the use of stratified multistage cluster sampling and (ii) the unequal probabilities of selection from target populations for sampled elements, often due to oversampling of key subgroups. To account for this complexity in the survey design, sampling weights were applied when estimating proportions and corresponding 95% confidence intervals (CIs) [23]. Descriptive analysis involved computing the sociodemographic characteristics of adolescent girls, presented in frequencies and percentages (see Table 2). Additionally, weighted percentages of anemia by different factors were computed (see Table 3).

**TABLE 2:** Sociodemographic and health related characteristics of adolescent girls in Africa (*N* = 41,662).

Predictors/factors	Frequency	Proportion
Anemia severity		
Not anemic	23,964	57.5
Mild anemia	8923	21.4
Moderate anemia	8137	19.5
Severe anemia	637	1.5
Over all prevalence of anemia	**41,662**	**42.3**
Residence		
Urban	16,941	40.7
Rural	24,721	59.3
Marital status		
Never married/single	33,686	80.9
Ever married	7974	19.1
Age at first marriage (*n* = 8174)		
< 15	1861	23.3
15–19	6115	76.7
Education status		
No education	6002	14.4
Primary	13,762	33.0
Secondary and higher	21,897	52.6
Wealth status		
Poorer	6627	15.9
Poor	7472	17.9
Middle	8471	20.3
Rich	9157	22.0
Richer	9934	23.8
Currently pregnant		
No	39,561	95.0
Yes	2101	5.0
Use of contraceptive		
Non user	37,025	89.0
Hormonal user	2727	6.6
Nonhormonal user	1850	4.4
Health insurance		
No	37,056	88.9
Yes	4605	11.1
Currently working		
No	28,168	67.6
Yes	13,494	32.4
Parity		
No birth	34,741	83.4
one or more births	6921	16.6
Body mass index		
Underweight	7800	18.7
Normal weight	29,392	70.5
Overweight/obese	4469	10.7
Mosquito net use		
No	18,346	56.2
Yes	14,300	43.8

*Note:* Frequencies and proportions are based on weighted survey data and may not sum to the overall sample size because of missing observations. The overall analytic sample size was *N* = 41,662; variable. Bold values indicate the overall magnitude of anemia.

**TABLE 3:** Association of anemia severity with explanatory variables (chi‐square analysis).

Variable	Design‐based *F*	*p* value
Type of place of residence	5.7183	< 0.001
Educational status	27.4887	< 0.001
Marital status	11.5599	< 0.001
Parity	6.9317	< 0.001
Currently pregnant	34.0168	< 0.001
Wealth index combined	8.3544	< 0.001
Working status	9.5587	< 0.001
BMI category	2.0465	0.0575
Covered by health insurance	8.7179	< 0.001
Contraceptive use	15.4807	< 0.001
Age at first marriage	0.2588	0.8530
Mosquito net use	3.4890	0.0153

*Note:* Analyses are survey‐weighted and account for the complex sampling design. Associations were assessed using the design‐based *F*‐adjusted chi‐square test. Statistical significance was set at *p* < 0.05. *N* = 41,662; *F* = design‐based *F* statistic.

Abbreviation: BMI, body mass index.

Given that adolescent girls residing within the same cluster may share similar characteristics. A multilevel modeling approach (by including survey clusters as random effects) was employed to account for within‐cluster correlation. Survey clusters were included as random effects to unobserved heterogeneity at the community level. A random intercept was specified at the cluster (primary sampling unit) level only; between‐country heterogeneity was therefore not explicitly partitioned as a separate random effect. This should be considered when interpreting the pooled estimates. A multilevel ordinal logistic regression model was fitted to examine the association between adolescent girls’ anemia status and explanatory variables, adjusting for potential confounders. Let *y*_*i* *j*_ denote the anemia levels for adolescent girls *i* in cluster *j*, is a categorized version of a latent continuous variable. Then, the model that takes into account the ordinal nature of the outcome (nonanemic, mild anemic, moderate anemic, and severe anemic) is formulates as follows:

(1) =logitPY_ij<KX_ijp_ij=β0k+xij′β+bj,

where *X* is a vector of different covariates presented in Table 2 with associated regression coefficients *β*. The random‐effect component of the model, *b*_*j*_ are mutually independent Gaussian random effects used to capture within‐cluster correlation [24].

The random‐effect component of the model captured within‐cluster correlation using mutually independent Gaussian random effects with mean 0 and variance $\sigma_{b}^{2}$. The fixed effects parameter in the model had a conditional interpretation, reflecting the consequence of changing the value of an explanatory variable for a given value of the random effect and the other fixed effects [25]. Both descriptive and inferential data analyses were done using Stata 18.0 software [26]. In this study, for statistical significance, considered Type‐I error alpha value is 0.05.

### 2.4. Sensitivity Analyses

To assess the robustness of the findings, several sensitivity analyses were conducted. First, the multilevel ordinal logistic regression model was re‐estimated by replacing age at first marriage with marital status to evaluate potential collinearity between these variables. Second, analyses were repeated after restricting the sample to nonpregnant adolescents. Third, anemia was alternatively modeled as any anemia vs none using a multilevel logistic regression model (see supporting information (available here)). The consistency of results across these analyses was examined to assess the stability of the main findings.

### 2.5. Ethical Consideration

This study exclusively involved secondary analysis of existing DHS data. The data collection procedures for the DHS adhered to all relevant guidelines and regulations. Ethical approval for the surveys was obtained from the Institutional Review Boards of each respective country. For adolescent girls under the age of 18, parental or guardian permission was required prior to participation in the study. The authors obtained authorization from the DHS program to use the data: https://dhsprogram.com/data/available-datasets.cfm.

## 3. Results

Table 2 presents the sociodemographic, economic, and health characteristics of 41,662 adolescent girls sampled from 25 various African countries. Most adolescents (59.3%) reside in rural areas. Regarding marital status, about 19.1% have ever been married. Among those who have been married, 23.3% married before the age of 15.

Nearly half of the adolescents (52.6%) had attained secondary and higher education, while 33% had completed primary education. Around 14.4% has no education. About 32.1% of adolescents were engaged in some form of employment (Table 2).

About 5% of adolescent girls were pregnant during the data collection period. A large proportion of the adolescents (89.0%) do not use any contraceptive method. while 6.6% used hormonal contraceptives and 4.4% used nonhormonal contraceptives. About 16.6% of adolescents had one or more children.

Most adolescents (88.9%) do not have health insurance. The majority of adolescents had a normal weight (70.5%), while 18.7% were underweight and 10.7% were overweight/obese. Most adolescents (56.2%) do not use mosquito nets.

Regarding anemia severity, about 21.4%, 19.5%, and 1.5% of adolescents had mild, moderate, and severe anemia, respectively.

Table **1** shows that the severity level of anemia among adolescent girls varied substantially across the 25 African countries. Overall, the proportion of adolescents with anemia ranged from 14.7% in Rwanda to 72.0% in Gabon. Countries with the highest prevalence of anemia included Gabon (72.0%), Mauritania (63.3%), Cote d’Ivoire (62.2%), Burkina Faso (57.6%), Benin (57.4%), Liberia (55.5%), and Mozambique (54.4), where more than half of adolescent girls were affected. In contrast, relatively lower prevalence was observed in Rwanda (14.7%), Ethiopia (19.9%), and Madagascar (27.8%).

Across most countries, mild and moderate anemia accounted for the majority of cases, while severe anemia was relatively uncommon. The prevalence of severe anemia was generally below 2% in most countries, although higher levels were observed in Mauritania (7.3%) and Gabon (3.5%). Moderate anemia contributed a substantial share of the burden, particularly in Gabon (36.2%), Mauritania (31.9%), Cote d’Ivoire (31.3%), and Burkina Faso (29.8%). Mild anemia was also common, ranging from about 10% to 30% across the countries.

Based on survey year, anemia prevalence was generally higher in surveys conducted between 2019–21 and 2021–23, as observed in countries such as Gabon (2019–21), Mauritania (2019–21), and Cote d’Ivoire (2021). In contrast, older surveys from 2016 to 17, including Ethiopia (2016), South Africa (2016), and Uganda (2016), reported lower prevalence, typically below 35%.

Table 3 presents a chi‐square analysis of the severity of anemia among African adolescent girls by various explanatory variables. The design‐based F test showed that place of residence, educational status, marital status, parity, pregnancy status, wealth index, employment status, health insurance coverage, contraceptive use, and mosquito net use were significantly associated with anemia severity (*p* < 0.05). However, body mass index and age at first marriage were not significantly associated with anemia severity.

Table 4 presents the ordinal logistic regression analysis of various factors associated with anemia severity among adolescent girls in Africa. The results are shown in terms of crude odds ratios (COR) and adjusted odds ratios (AOR), along with their 95% CI.

**TABLE 4:** Bivariate and multivariable analysis of anemia severity among adolescent girls in Africa.

Factors	COR (95% CI)	AOR (95% CI)
Residence		
Urban	1	1
Rural	0.92 (0.86–0.98)^∗∗^	0.98 (0.86–1.12)
Marital status		
Never married/single	1	1
Ever married	1.27 (1.19–1.35)^∗∗^	
Age at first marriage (*n* = 8298)		
< 15	1	1
15–19	0.97 (0.85–1.10)^∗^	0.96 (0.86–1.07)
Education status		
No education	1	1
Primary	0.62 (0.57–0.67)^∗∗^	**0.84 (0.75-0.94)** ^∗^
Secondary and higher	0.69 (0.64–0.74)^∗∗^	**0.86 (0.75-0.98)** ^∗^
Wealth status		
Poorer	1	1
Poor	0.91 (0.84–0.98)^∗∗^	0.99 (0.88–1.14)
Middle	0.85 (0.79–0.93)^∗∗^	0.87 (0.76–1.01)
Rich	0.77 (0.70–0.84)^∗^	**0.77 (0.66-0.91)** ^∗^
Richer	0.71 (0.65–0.77)^∗^	**0.72 (0.59-0.89)** ^∗^
Currently pregnant		
No	1	1
Yes	1.63 (1.47–1.81)^∗∗^	1.05 (0.93–1.19)
Use of contraceptive		
No	1	1
Hormonal user	0.63 (0.57–0.70)^∗∗^	**0.44 (0.38-0.51)** ^∗^
Nonhormonal user	1.08 (0.97–1.20)	0.82 (0.64–1.06)
Health insurance		
No	1	1
Yes	0.81 (0.74–0.88)^∗∗^	0.99 (0.79–1.24)
Currently working/occupation		
No	1	1
Yes	0.92 (0.88–0.97)^∗^	**0.86 (0.78-0.94)** ^∗^
Parity		
No	1	1
One or more births	1.15 (1.08–1.23)^∗∗^	**0.85 (0.76-0.95)** ^∗^
Body mass index		
Underweight	1	1
Normal weight	0.96 (0.90–1.02)	**0.84 (0.73-0.97)** ^∗^
Overweight/obese	0.90 (0.82–0.99)^∗∗^	**0.75 (0.62-0.91)** ^∗^
Use of mosquito net		
No	1	1
Yes	1.09 (1.05–1.14)^∗∗^	0.98 (0.89–1.07)
Intraclass correlation coefficient (ICC)		0.0195
Random‐intercept variance (PSU level):		0.0655

*Note:* Reference categories are indicated by COR/AOR = 1. The overall analytic sample was *N* = 41,662, with variable‐specific sample sizes varying because of missing data. Statistical significance: ^∗^ *p* < 0.2; ^∗∗^ *p* < 0.05. Bold values indicate *p* < 0.05 in the multivariable analysis.

Abbreviations: AOR, adjusted odds ratio; CI, confidence interval; COR, crude odds ratio; ICC, intraclass correlation coefficient; PSU, primary sampling unit.

^∗^Analyses were survey‐weighted and accounted.

Adolescent girls living in rural areas had a slightly lower odds of anemia compared to those in urban areas (COR: 0.93, 95% CI: 0.87–0.99). However, this association is not significant after adjusting for other factors (AOR: 0.98, 95% CI: 0.86–1.12). Adolescent girls who had ever married showed higher odds of anemia compared to their never‐married/single counterparts (COR: 1.27, 95% CI: 1.19–1.36). However, this variable was omitted from the multivariable analysis due to multicollinearity issues.

Education appears protective against anemia severity. Compared to adolescents with no education, those with primary (AOR: 0.84, 95% CI: 0.75–0.94) and secondary and above (education [AOR: 0.86, 95% CI: 0.75–0.98] had lower odds of anemia). Adolescents from rich and richer households have significantly lower odds of anemia severity compared to those from poor households (AOR: 0.77 95% CI: 0.65–0.90 and AOR: 0.72, 95% CI: 0.58–0.89). Regarding occupational status, adolescents who were working had significantly lower odds of anemia severity compared with those who were not working (AOR = 0.85, 95% CI: 0.78–0.94).

Pregnancy was associated with higher odds of anemia severity (COR: 1.63, 95% CI: 1.47–1.81) compared to those who were not pregnant. However, this association was not significant after adjustment of other factors (AOR: 1.05, 95% CI: 0.93–1.19). Using hormonal contraceptives was linked to significantly lower odds of anemia severity (AOR: 0.44, 95% CI 0.38–0.51) compared with adolescents who did not use any contraceptive methods. However, the use of nonhormonal contraceptives was not statistically significantly associated with anemia severity compared with nonusers (AOR: 0.82, 95% CI: 0.64–1.06). After adjusting for other factors, adolescents who had given birth to one or more children had lower odds of anemia severity compared to those with no children (AOR: 0.85, 95% CI: 0.76–0.95).

Having health insurance is associated with lower odds of anemia severity in the bivariate analysis (COR: 0.81, 95% CI: 0.74–0.88). However, this association disappeared in the multivariate analysis (AOR: 0.99, 95% CI: 0.791–1.24). Regarding nutritional status, adolescents with normal weight and those who were overweight/obese had significantly lower odds of anemia severity compared with those who were underweight (AOR: 0.84, 95% CI: 0.73–0.97 and AOR: 0.75, 95% CI: 0.62–0.91).

The multivariable analysis revealed that 1.95% of the variation in anemia severity among adolescent girls was due to differences at the cluster/community level (ICC = 0.0195, *p* < 0.05). The estimated random‐intercept variance at the cluster level was 0.0655, corresponding to an ICC of 0.0195. This indicates that approximately 1.95% of the total latent‐scale variation in anemia severity was attributable to differences between clusters or communities, while the remaining 98.05% of the variation occurred within clusters, at the individual level.

## 4. Discussion

This study aimed to investigate the severity and determinants of anemia among adolescent girls in 25 African countries. The analysis utilized both bivariate and multivariable logistic regression to identify the relationship between various sociodemographic, economic, and health‐related factors with the likelihood of anemia severity. The study investigated that the overall prevalence of anemia among adolescent girls in Africa was 42.3% (95% CI: 41.96–44.01). According to the WHO, anemia is categorized based on prevalence cut‐off values that indicate its public health problem. If the prevalence of anemia is below 5%, it is considered not to be a public health problem. A prevalence of 5%–19% is classified as a mild public health problem, while a prevalence of 20%–39% is considered a moderate public health problem. When the prevalence reaches 40% or higher, anemia is considered a severe public health problem [27]. Given that the prevalence of anemia among adolescent girls in Africa exceeds this threshold at 42.3%, it is classified as a severe public health problem in the region.

In line with our previous work published as a preprint on Research Square [20], the prevalence of anemia in Africa was 43.6% (95% CI: 42.97–44.21) which is higher than the studies conducted in Indonesia (14.3%) [28], India (20%) [12], Nepal (31%) [29], Ethiopia (23%) [8], and Kenya (28.9%) [30], but lower than in Pakistan (47.9%) [31], Tanzania (53.3%) [9], and Ghana (50.3%) [32]. Even within this study, the severity of anemia among adolescent girls varied significantly across different African countries. The prevalence ranged from 14.7% in Rwanda to 72% in Gabon. This wide range in anemia prevalence reflects differences in factors such as religion, cultural and dietary practices, access to iron‐rich foods, socioeconomic conditions, health system, and the effectiveness of public health interventions in these countries [33–35].

Furthermore, out of the 25 African countries included in this study, 17 of them (68%) had a prevalence of anemia above 40% among adolescent girls, which is alarming. This indicates that in the majority of African countries, the magnitude of anemia among this population is a substantial public health problem that requires urgent attention. Addressing this public health challenge will require a comprehensive, multifaceted approach tailored to the specific needs and contexts of each country. Interventions may include improving dietary diversity and nutrient intake, implementing supplementation programs, providing health education, and enhancing access to healthcare services to adolescent girls [36, 37].

Our multivariable analysis found that adolescents’ educational level, wealth status, occupational status, parity, contraceptive use, and nutritional status were statistically significant with anemia severity.

In our previous work published as a preprint on Research Square, which included data from 24 African countries, the outcome variable was measured dichotomously (anemic vs. nonanemic). The multivariable analysis of this previous work reported adolescents’ educational level, household wealth status, pregnancy status, contraceptive use, nutritional status, and mosquito net utilization as statistically significant predictors of anemia [20].

In the current revised analysis, based on data from 25 African countries, the outcome variable was re‐categorized into four ordered levels of anemia severity and analyzed using an ordinal logistic regression model. In addition, the dataset was updated to include countries with more recent DHS data (such as Côte d’Ivoire, Lesotho, Madagascar, and Mauritania) and to exclude countries with older datasets (more than 10 years), including Equatorial Guinea and Ivory Coast.

Compared to the previous findings, some variables (educational level, wealth status, contraceptive use, and nutritional status) remained consistently significant, indicating their robust association with anemia across different model specifications. However, pregnancy status and mosquito net use were no longer significant in the current study, while occupational status and parity emerged as new significant predictors. These differences can be justified by two key factors. First, the shift from a dichotomous to an ordinal outcome variable allowed for a more nuanced assessment of anemia severity, which can alter the strength and significance of associations. Second, changes in the dataset, specifically the inclusion of more recent country data and exclusion of outdated datasets, likely influenced the observed associations due to differences in population structure, health service access, and epidemiological patterns across countries and time.

Education emerged as a crucial protective factor against anemia. Adolescent girls with primary and secondary education levels showed significantly lower odds of anemia severity compared to those with no education which was supported by other studies done in sub‐Saharan Africa [13], West and Central Africa [38], and Ethiopia [15]. This finding indicates that education is crucial in enhancing health literacy, nutritional awareness, and overall health practices, which can considerably lower the risk of anemia. Education likely equips adolescents with the knowledge and resources needed to make health decisions, leading to improved nutritional choices and proactive health‐seeking behaviors [39, 40]. Unsurprisingly and similar to other study findings [41, 42], our study found that adolescents from rich and richer households also had lower odds of anemia compared to those from poor households. This suggests that economic resources improve access to nutrition and healthcare services, both of which are essential for preventing anemia. Families with higher incomes can afford more diverse, nutritious diets, healthcare services, and supplements, all of which help to prevent anemia.

This study revealed that the use of hormonal contraceptives was significantly associated with lower odds of anemia which was supported by other studies [43, 44]. This could be justified by the fact that adolescents who use hormonal contraceptives can prevent complications related to early pregnancy and childbirth, which may reduce the prevalence of anemia caused by recurrent blood loss. Another plausible justification is that the use of hormonal contraceptives can minimize menstrual bleeding and decrease their susceptibility to anemia [45, 46].

Interestingly, this study found that adolescent girls with one or more birth histories had significantly lower odds of anemia severity compared with those who had no birth history. This finding contradicts several previous studies [10, 47, 48], which reported that higher parity increases the risk of anemia due to repeated pregnancies, increased iron requirements, and depletion of maternal iron stores. The difference observed in the present study may be explained by greater exposure to maternal health services among adolescents who have already given birth. Parous adolescents are more likely to have contact with antenatal care services, where they may receive iron–folic acid supplementation, nutrition counseling, deworming treatment, and hemoglobin screening, all of which contribute to improved hemoglobin status [49, 50]. In contrast, adolescents with no birth history may have limited interaction with health services and fewer opportunities to receive preventive nutritional interventions, which may increase their susceptibility to anemia. Therefore, the apparent protective association of birth history with anemia in this study likely reflects increased healthcare utilization and nutritional support during pregnancy rather than a biological protective effect of parity.

Adolescent girls who were normal weight and overweight/obese showed significantly lower odds of anemia compared to those who were underweight. Our result was consistent with the study conducted in Pakistan [51], in Bangladesh [52], and in Ethiopia [15]. This suggests that normal body weight, potentially linked to better micronutrient status, may confer some protection against anemia. On the other hand, underweighted adolescents have more chances to be deficient in micronutrients which may result in an increased risk of anemia. However, it is important to note that while obesity might reduce the risk of anemia, it is not an indicator of overall health and poses other health risks. Nevertheless, studies done in Korea [53] and in Morocco [54] reported a higher probability of anemia among obese adolescents. The possible explanation might be obesity is often associated with chronic inflammation, which can affect iron metabolism. Inflammatory cytokines can inhibit iron absorption and utilization, leading to anemia, despite adequate or elevated iron stores. Obese individuals may have diets that are high in calories but low in essential nutrients, including iron [55]. Furthermore, overweight/obese individuals have a higher blood volume, which may dilute the concentration of hemoglobin and ferritin, potentially masking the presence of anemia when only hemoglobin levels are measured. The relationship between overweight/obesity and anemia among adolescents is multifaceted. It underscores the importance of considering various factors, including micronutrient status, dietary quality, and inflammation, when evaluating the risk of anemia. Further research is needed to clarify these associations and inform public health strategies to improve the nutritional status of adolescent girls.

This study has some limitations to consider. (a) The cross‐sectional nature of DHS data prevents establishing temporal relationships or causal effects between the identified factors and anemia severity. (b) Due to the limited variables available in this secondary data, several important determinants of anemia were not available across all included DHS datasets. These include dietary iron intake and dietary diversity, food insecurity, malaria and helminth infection status, inflammatory biomarkers, iron stores/ferritin, recent illness, and other biological and environmental determinants. Therefore, residual confounding cannot be excluded. Despite these limitations, our study included important proxy variables for food security, such as wealth status, as well as other sociodemographic and economic factors. Using a continentally representative large sample, the results from this analysis may be generalized to African adolescent girls aged 15–19 years.

## 5. Conclusion

Anemia among adolescent girls in Africa remains a severe public health problem, with a pooled prevalence of 42.3%, exceeding the threshold defined by the WHO. Educational level, wealth status, occupational status, contraceptive use, parity, and nutritional status were significant determinants of anemia severity. These findings highlight the importance of improving girls’ education and socioeconomic conditions. Furthermore, enhancing adolescents’ utilization of health services may help reduce anemia by improving access to reproductive health care and nutritional support.

**Nomenclature**
AOR: Adjusted odds ratio
BMI: Body mass index
CI: Confidence interval
COR: Crude odds ratio
DHS: Demographic health survey

## Author Contributions

Fentanesh Nibret Tiruneh designed the study, drafted, and reviewed the manuscript. Bedilu Alamirie Ejigu wrote the method section and performed data management and analysis. Getnet Bogale Begashaw runs the revised analysis and edits the revised version of the manuscript. The corresponding author (Fentanesh Nibret Tiruneh) had full access to all of the data in this study and takes complete responsibility for the integrity of the data and the accuracy of the data analysis.

## Funding

The authors have nothing to report.

## Disclosure

An earlier version of this manuscript was previously made available as a preprint on Research Square (https://www.researchsquare.com/article/rs-5004469/v1). The authors confirm that no individuals or third‐party services contributed to this research. A preprint version of this manuscript was previously posted on Research Square (https://doi.org/10.21203/rs.3.rs-5004469/v1). The current submission has been revised and expanded, and it is not under consideration elsewhere. Compared to the preprint version, this manuscript includes updated analyses, improved methodology description, and revised interpretation of findings. All authors have read and approved the final version of the manuscript.

## Ethics Statement

The study analyzed existing DHS data, adhering to ethical guidelines, with informed consent obtained from all participants and necessary permissions secured. The authors obtained permission from the DHS program to use the data.

## Consent

The authors have nothing to report.

## Conflicts of Interest

The authors declare no conflicts of interest.

## Supporting Information

Additional supporting information can be found online in the Supporting Information section.

## Supporting information


**Supporting Information** Additional analyses are provided in the supporting information, including sensitivity analyses examining the robustness of the findings using binary anemia status, marital status, and a nonpregnant subsample, as well as the Stata code used for the analyses.

## Data Availability

The data that support the findings of this study are available in DHS at https://dhsprogram.com/data/available-datasets.cfm. These data were derived from the following resources available in the public domain: https://dhsprogram.com/data/available-datasets.cfm, https://dhsprogram.com/data/available-datasets.cfm).
