# Supplementary material for: Severity and Determinants of Anemia Among Adolescent Girls in Africa: A Multicountry and Multilevel Analysis of 25 Countries
Source: Anemia. 2026 Oct 18;2026:1066665. doi: 10.1155/anem/1066665 (PMC13678286; doi:10.1155/anem/1066665)
Supplement: Supplementary file 1 [file ANEM-2026-1066665-s001.zip › Sensitivity_Model_BinaryAnemia.docx]

Sensitivity analysis 3: Mixed-effects logistic regression with binary anemia outcome

| anemia_bin | Odds ratio | Std. err. | z | P>\|z\| | [95% conf. interval] | |
| --- | --- | --- | --- | --- | --- | --- |
| ur |  |  |  |  |  |  |
| rural | .9729578 | .0698733 | -0.38 | 0.703 | .8452101 | 1.120014 |
|  |  |  |  |  |  |  |
| education |  |  |  |  |  |  |
| Primary education | .8801749 | .0533873 | -2.10 | 0.035 | .7815182 | .9912857 |
| Secondary or higher | .9078816 | .0660249 | -1.33 | 0.184 | .7872747 | 1.046965 |
|  |  |  |  |  |  |  |
| Parity |  |  |  |  |  |  |
| One or more births | .8769929 | .0509158 | -2.26 | 0.024 | .7826681 | .9826854 |
|  |  |  |  |  |  |  |
| Pregnant |  |  |  |  |  |  |
| yes | 1.005838 | .068215 | 0.09 | 0.932 | .880644 | 1.14883 |
|  |  |  |  |  |  |  |
| wealth |  |  |  |  |  |  |
| poorer | 1.017871 | .0717297 | 0.25 | 0.802 | .8865607 | 1.168631 |
| middle | .8872291 | .0659187 | -1.61 | 0.107 | .7669973 | 1.026308 |
| richer | .788163 | .0687167 | -2.73 | 0.006 | .6643596 | .935037 |
| richest | .7638727 | .0866806 | -2.37 | 0.018 | .6115482 | .9541382 |
|  |  |  |  |  |  |  |
| occupation |  |  |  |  |  |  |
| yes | .8933349 | .0468872 | -2.15 | 0.032 | .8060064 | .9901251 |
|  |  |  |  |  |  |  |
| BMIclass |  |  |  |  |  |  |
| Normal | .8906916 | .0709804 | -1.45 | 0.146 | .761893 | 1.041264 |
| Overweight/obese | .7841147 | .0812729 | -2.35 | 0.019 | .6399606 | .9607402 |
|  |  |  |  |  |  |  |
| Hinsurance |  |  |  |  |  |  |
| yes | 1.066462 | .1307224 | 0.52 | 0.600 | .8387043 | 1.35607 |
|  |  |  |  |  |  |  |
| mcp |  |  |  |  |  |  |
| Hormonal user | .4341117 | .0342341 | -10.58 | 0.000 | .3719423 | .5066725 |
| Non-hormonal user | .8017024 | .1100426 | -1.61 | 0.107 | .612599 | 1.04918 |
|  |  |  |  |  |  |  |
| agemarriage |  |  |  |  |  |  |
| 15–19 years | .9401731 | .0578391 | -1.00 | 0.316 | .8333783 | 1.060653 |
|  |  |  |  |  |  |  |
| mnet |  |  |  |  |  |  |
| Use of net | 1.002724 | .0517978 | 0.05 | 0.958 | .9061727 | 1.109563 |
| _cons | 1.68157 | .2115612 | 4.13 | 0.000 | 1.314086 | 2.151821 |
| v021 |  |  |  |  |  |  |
| var(_cons) | .0483662 | .0252246 |  |  | .0174025 | .1344228 |
