# Supplementary material for: Severity and Determinants of Anemia Among Adolescent Girls in Africa: A Multicountry and Multilevel Analysis of 25 Countries
Source: Anemia. 2026 Oct 18;2026:1066665. doi: 10.1155/anem/1066665 (PMC13678286; doi:10.1155/anem/1066665)
Supplement: Supplementary file 1 [file ANEM-2026-1066665-s001.zip › Sensitivity_Model_MaritalStatus.docx]

Sensitivity analysis 1: Model including marital status instead of age at first marriage

| anemia_ord | Odds ratio | Std. err. | z | P>\|z\| | [95% conf. interval] | |
| --- | --- | --- | --- | --- | --- | --- |
| ur |  |  |  |  |  |  |
| rural | 0.812 | 0.022 | -7.74 | 0.000 | 0.771 | 0.856 |
|  |  |  |  |  |  |  |
| education |  |  |  |  |  |  |
| Primary education | 0.721 | 0.024 | -9.74 | 0.000 | 0.675 | 0.770 |
| Secondary or higher | 0.794 | 0.027 | -6.71 | 0.000 | 0.742 | 0.849 |
|  |  |  |  |  |  |  |
| Parity |  |  |  |  |  |  |
| One or more births | 1.063 | 0.039 | 1.68 | 0.093 | 0.990 | 1.142 |
|  |  |  |  |  |  |  |
| Pregnant |  |  |  |  |  |  |
| yes | 1.374 | 0.069 | 6.35 | 0.000 | 1.246 | 1.516 |
|  |  |  |  |  |  |  |
| wealth |  |  |  |  |  |  |
| poorer | 0.876 | 0.031 | -3.74 | 0.000 | 0.817 | 0.939 |
| middle | 0.843 | 0.030 | -4.83 | 0.000 | 0.787 | 0.904 |
| richer | 0.715 | 0.027 | -8.93 | 0.000 | 0.664 | 0.769 |
| richest | 0.568 | 0.023 | -13.87 | 0.000 | 0.525 | 0.616 |
|  |  |  |  |  |  |  |
| occupation |  |  |  |  |  |  |
| yes | 0.850 | 0.020 | -7.02 | 0.000 | 0.812 | 0.889 |
|  |  |  |  |  |  |  |
| BMIclass |  |  |  |  |  |  |
| Normal | 0.919 | 0.026 | -2.96 | 0.003 | 0.869 | 0.972 |
| Overweight/obese | 0.835 | 0.035 | -4.30 | 0.000 | 0.769 | 0.907 |
|  |  |  |  |  |  |  |
| Hinsurance |  |  |  |  |  |  |
| yes | 0.787 | 0.026 | -7.24 | 0.000 | 0.738 | 0.840 |
|  |  |  |  |  |  |  |
| mcp |  |  |  |  |  |  |
| Hormonal user | 0.606 | 0.029 | -10.61 | 0.000 | 0.553 | 0.665 |
| Non-hormonal user | 1.006 | 0.055 | 0.11 | 0.912 | 0.905 | 1.119 |
|  |  |  |  |  |  |  |
| mstatus |  |  |  |  |  |  |
| Ever married | 1.089 | 0.040 | 2.33 | 0.020 | 1.014 | 1.170 |
|  |  |  |  |  |  |  |
| mnet |  |  |  |  |  |  |
| Use of net | 0.992 | 0.022 | -0.39 | 0.697 | 0.950 | 1.035 |
| /cut1 | -0.532 | 0.049 |  |  | -0.629 | -0.435 |
| /cut2 | 0.513 | 0.050 |  |  | 0.416 | 0.610 |
| /cut3 | 3.391 | 0.064 |  |  | 3.265 | 3.518 |
| v021 |  |  |  |  |  |  |
| var(_cons) | 0.014 | 0.005 |  |  | 0.007 | 0.028 |
