# Supplementary material for: Severity and Determinants of Anemia Among Adolescent Girls in Africa: A Multicountry and Multilevel Analysis of 25 Countries
Source: Anemia. 2026 Oct 18;2026:1066665. doi: 10.1155/anem/1066665 (PMC13678286; doi:10.1155/anem/1066665)
Supplement: Supplementary file 1 [file ANEM-2026-1066665-s001.zip › Sensitivity_Model_NonPregnant.docx]

Sensitivity analysis 2: Model restricted to non-pregnant adolescents

| anemia_ord | Odds ratio | Std. err. | z | P>\|z\| | [95% conf. interval] | |
| --- | --- | --- | --- | --- | --- | --- |
| ur |  |  |  |  |  |  |
| rural | 1.064 | 0.082 | 0.80 | 0.423 | 0.914 | 1.239 |
|  |  |  |  |  |  |  |
| education |  |  |  |  |  |  |
| Primary education | 0.831 | 0.054 | -2.86 | 0.004 | 0.732 | 0.943 |
| Secondary or higher | 0.781 | 0.061 | -3.16 | 0.002 | 0.670 | 0.910 |
|  |  |  |  |  |  |  |
| Parity |  |  |  |  |  |  |
| One or more births | 0.901 | 0.056 | -1.68 | 0.093 | 0.798 | 1.017 |
|  |  |  |  |  |  |  |
| wealth |  |  |  |  |  |  |
| poorer | 0.993 | 0.074 | -0.09 | 0.928 | 0.858 | 1.150 |
| middle | 0.871 | 0.069 | -1.74 | 0.083 | 0.745 | 1.018 |
| richer | 0.805 | 0.075 | -2.33 | 0.020 | 0.671 | 0.966 |
| richest | 0.787 | 0.097 | -1.94 | 0.052 | 0.618 | 1.002 |
|  |  |  |  |  |  |  |
| occupation |  |  |  |  |  |  |
| yes | 0.872 | 0.049 | -2.45 | 0.014 | 0.781 | 0.973 |
|  |  |  |  |  |  |  |
| BMIclass |  |  |  |  |  |  |
| Normal | 0.831 | 0.066 | -2.33 | 0.020 | 0.711 | 0.971 |
| Overweight/obese | 0.776 | 0.084 | -2.33 | 0.020 | 0.627 | 0.960 |
|  |  |  |  |  |  |  |
| Hinsurance |  |  |  |  |  |  |
| yes | 1.024 | 0.133 | 0.18 | 0.855 | 0.794 | 1.320 |
|  |  |  |  |  |  |  |
| mcp |  |  |  |  |  |  |
| Hormonal user | 0.431 | 0.034 | -10.75 | 0.000 | 0.370 | 0.503 |
| Non-hormonal user | 0.828 | 0.109 | -1.43 | 0.152 | 0.639 | 1.072 |
|  |  |  |  |  |  |  |
| agemarriage |  |  |  |  |  |  |
| 15–19 years | 0.977 | 0.063 | -0.36 | 0.716 | 0.861 | 1.108 |
|  |  |  |  |  |  |  |
| mnet |  |  |  |  |  |  |
| Use of net | 0.968 | 0.054 | -0.59 | 0.553 | 0.868 | 1.079 |
| /cut1 | -0.553 | 0.132 |  |  | -0.812 | -0.294 |
| /cut2 | 0.474 | 0.133 |  |  | 0.214 | 0.734 |
| /cut3 | 3.363 | 0.162 |  |  | 3.045 | 3.681 |
| v021 |  |  |  |  |  |  |
| var(_cons) | 0.104 | 0.033 |  |  | 0.056 | 0.193 |
